# Do critically ill patients with AKI benefit from furosemide? Further real-word evidence from a large multi-center database

**DOI:** 10.1186/s13054-020-02905-7

**Published:** 2020-05-25

**Authors:** Shaowei Gao, Lu Yang, Zhongxing Wang

**Affiliations:** Department of Anesthesia, the First Affiliated Hospital of Sun Yat-sen University, 58 Zhongshan 2nd Road, Guangzhou, 510080 China

We are interested in the recently published article about the effect of furosemide on the outcomes of patients with acute renal injury (AKI) [1], because the result seems against to the current guidelines [2].

AKI is commonly faced by the intensivists and its effects on mortality have drawn great attention recently. Zhao and his colleagues explored the effect of furosemide on the outcomes of critically ill patients with AKI in a real-word dataset, the MIMIC III database. They found the furosemide administration was associated with better short-term survival, especially in the AKI^UO^ stages 2–3. However, the dataset they used in the analysis is single-center.

We notice that another real-world database, the eICU database, which has larger size of data (200,859 patients) and more contributing centers (335 units at 208 hospitals), has been available to the world [3]. We reproduced the main analysis of Zhao’s study in the eICU database to give more evidence for the real-world research. The main results for outcomes are presented in Table 1.

**Table 1:** Association between furosemide use and clinical outcomes in critically ill patients with acute kidney injury

Outcomes	Non-diuretics	Furosemide	*p* value	HR (95% CI)
Pre-matched cohorts	48,495	11,256		
Primary				
In-hospital mortality, *n* (%)^a^	5048 (10.4)	1327 (11.8)	0.18	0.96 (0.90–1.02)
In-hospital mortality, *n* (%)^b^	5048 (10.4)	1327 (11.8)	0.98	1.00 (0.93–1.07)
Secondary				
Recovery of renal function, *n* (%)^b^	30,031 (61.9)	7105 (63.1)	< 0.001	1.10 (1.05–1.14)
Length of ICU stay, mean (SD)^c^	4.1 (5.0)	4.7 (5.2)	< 0.001	1.53 (1.35–1.73)
Length of hospital stay, mean (SD)^c^	10.3 (9.0)	11.1 (8.7)	< 0.001	1.46 (1.18–1.80)
Post-matched cohorts	11,256	11,256		
Primary				
In-hospital mortality, *n* (%)^a^	1415 (12.6)	1327 (11.8)	0.03	0.92 (0.85–0.99)
In-hospital mortality, *n* (%)^b^	1415 (12.6)	1327 (11.8)	0.49	0.97 (0.89–1.06)
Secondary				
Recovery of renal function, *n* (%)^b^	6791 (60.3)	7105 (63.1)	< 0.001	1.12 (1.06–1.19)
Length of ICU stay, mean (SD)^c^	4.3 (4.8)	4.7 (5.2)	< 0.001	1.41 (1.20–1.65)
Length of hospital stay, mean (SD)^c^	10.7 (8.9)	11.1 (8.7)	< 0.001	1.55 (1.19–2.00)

^a^Cox proportional hazard regression was used to adjust the confounding variables

^b^Logistic regression was used to adjust the confounding variables

^c^Linear regression was used to adjust the confounding variables

The confounding variables include age, gender, ethnicity, admission type, comorbidities, mechanical ventilation use, vasopressor use, fluid balance first day, serum creatinine, eGFR, and APCHE IV predicted hospital mortality. Sensitivity analysis was conducted by selecting a part of all variables by clinical experts, and the *p* value never changed from < 0.05 to > 0.05 or from > 0.05 to < 0.05, which presented robust

*Abbreviations*: *HR* hazard ratio, *CI* confidence interval, *ICU* intensive care unit, *SD* standard deviation

Some results are similar with Zhao’s study [1]: the furosemide group has a higher recovery rate and longer hospital and ICU stay. However, a reduction in in-hospital mortality for the furosemide group only exists in the dataset after propensity score matching (PSM) and under the method of Cox regression (HR 0.92; 95% CI 0.85–0.99; *p* = 0.03). For subgroup analysis, furosemide reduces mortality only in AKI^SCR^ stages 2 and 3, but not in any of AKI or AKI^UO^ stages.

Up to now, the main viewpoint is that diuretics including furosemide do not improve survival of AKI patients. The protective effect by improved renal function observed in some studies [1, 4] (include Zhao’s and ours) may be covered by the adverse events, such as electrolyte abnormalities [5]. Our study supports no benefit for furosemide on in-hospital mortality of AKI patients, despite a significant *p* value in the post-PSM dataset under the method of Cox regression (Table 1). Most of the studies use logistic regression to evaluate in-hospital mortality, because longer hospital stay for acute condition is not like longer follow-up time in chronic condition and may implicate bad outcomes. However, since the in-hospital mortality nearly doubles in the non-diuretic group comparing to the furosemide group in Zhao’s study (21.7% vs 12.7%), the debate of this issue still exists and needs more well-designed research.

## Authors’ response

### Response to the letter entitled “Do critically ill patients with AKI benefit from furosemide? Further real-word evidence from a large multi-center database”

Guang-ju Zhao, Chang Xu, Zhong-qiu Lu

We appreciate Dr. Wang and his colleagues for their comments on our recent article related to the association between furosemide use and outcomes in critically ill patients with AKI [1]. Using the eICU database, they found that furosemide use was associated with increased renal function recovery rate but not reduced in-hospital mortality. MIMIC III is a single-center database which contains ICU patient data between 2001 and 2012, while the eICU database covers patients who were admitted to 208 centers in 2014 and 2015. The inconsistent results may be due to the different treatment strategies of AKI in the two periods. Nevertheless, many previous studies also showed conflicting results, the potential reasons for this is worth exploring.

First, the protective effect of diuretic on AKI is at least partly mediated by fluid balance [1, 6]. So, the diversities of volume status among different AKI cohorts need to be considered when interpreting the conflicting results. Second, it has been noticed that the association between increased risk of death and furosemide use was more frequently reported in cohorts with higher SCr. So, in addition to the increased levels of SCr to baseline, the values of SCr and eGFR may also contribute to differences in treatment outcomes. Finally, the start time of diuretics may also determine the effect of them on the outcomes of AKI. In our cohort, there were 1591 AKI patients who received furosemide treatment 48 h after admission, and the mortality of them was similar to those without diuretic treatment (22.3% versus 21.7%, *p* > 0.05).

Recently, more and more new sub-classes of AKI with different clinical profiles, including mortality, the speed of renal function recovery, and fluid and furosemide responsiveness, have been recognized [7–9]. In selected AKI patients, some real-world studies (including Dr. Wang’s and ours) and multi-center prospective cohort studies have illustrated that furosemide use was associated with improved renal function recovery rate and (or) reduced mortality. Nevertheless, further high-quality study is needed to identify the sub-classes of AKI that can benefit from furosemide treatment.

## Data Availability

The datasets generated and/or analyzed during the current study are available in the eICU database (https://eicu-crd.mit.edu/).

## Abbreviations

AKI: Acute renal injury
PSM: Propensity score match
ICU: Intensive care unit
HR: Hazard ratio
CI: Confidence interval
